# Sex differences in transthyretin cardiac amyloidosis

**DOI:** 10.1007/s10741-023-10339-w

**Published:** 2023-08-11

**Authors:** Alberto Aimo, Giorgia Panichella, Manuel Garofalo, Simone Gasparini, Chiara Arzilli, Vincenzo Castiglione, Giuseppe Vergaro, Michele Emdin, Silvia Maffei

**Affiliations:** 1 Interdisciplinary Center for Health Sciences, Scuola Superiore Sant’Anna, Pisa, Italy; 2 Fondazione Toscana Gabriele Monasterio, Pisa, Italy; 3 Department of Experimental and Clinical Medicine, University of Florence, Florence, Italy; 4 Paediatric Neurology Unit and Laboratories, Neuroscience Department, Meyer Children’s Hospital IRCCS, Florence, Italy

**Keywords:** Transthyretin, ATTR, Cardiac amyloidosis, Sex, Diagnosis, Prognosis, Treatment

## Abstract

Transthyretin cardiac amyloidosis (ATTR-CA) is a progressive disease characterized by the deposition of abnormal transthyretin protein fibrils in the heart, leading to cardiac dysfunction. Recent evidence suggests that sex differences may play a significant role in various steps of ATTR-CA, including clinical presentation, diagnostic challenges, disease progression, and treatment outcomes. ATTR-CA predominantly affects men, whereas women are older at presentation. Women generally present with a history of heart failure with preserved ejection fraction and/or carpal tunnel syndrome. When indexed, left ventricular (LV) wall thickness is equal, or even increased, than men. Women also have smaller LV cavities, more preserved ejection fractions, and apparently a slightly worse right ventricular and diastolic function. Given the under-representation on women in clinical trials, no data regarding sex influence on the treatment response are currently available. Finally, it seems there are no differences in overall prognosis, even if premenopausal women may have a certain level of myocardial protection. Genetic variations, environmental factors, and hormonal changes are considered as potential contributors to observed disparities. Understanding sex differences in ATTR-CA is vital for accurate diagnosis and management. By considering these differences, clinicians can improve diagnostic accuracy, tailor treatments, and optimize outcomes for both sexes with ATTR-CA.

Transthyretin cardiac amyloidosis (ATTR-CA) is a progressive and life-threatening disease characterized by the deposition of abnormal transthyretin (TTR) protein fibrils within the heart tissue [1]. ATTR-CA predominantly affects the elderly population and is associated with significant morbidity and mortality [1]. While extensive research has focused on understanding the pathophysiology, diagnosis, and management of ATTR-CA, an emerging body of evidence suggests that sex differences may play a crucial role in the disease’s clinical manifestations, progression, and outcomes [2, 3]. Exploring these differences may not only improve the current understanding of the disease but also provide insights into developing personalized and sex-specific management strategies for patients with ATTR-CA.

In this review, we aim to examine and summarize the existing literature surrounding sex differences in ATTR-CA. We will delve into the epidemiological aspects of the disease, assessing the prevalence, incidence, and age at onset in both male and female populations. Furthermore, we will explore the differences in clinical manifestations, disease progression, and prognosis, as well as the possible mechanisms of sex differences in ATTR-CA (Fig. 1).

**Fig. 1:**
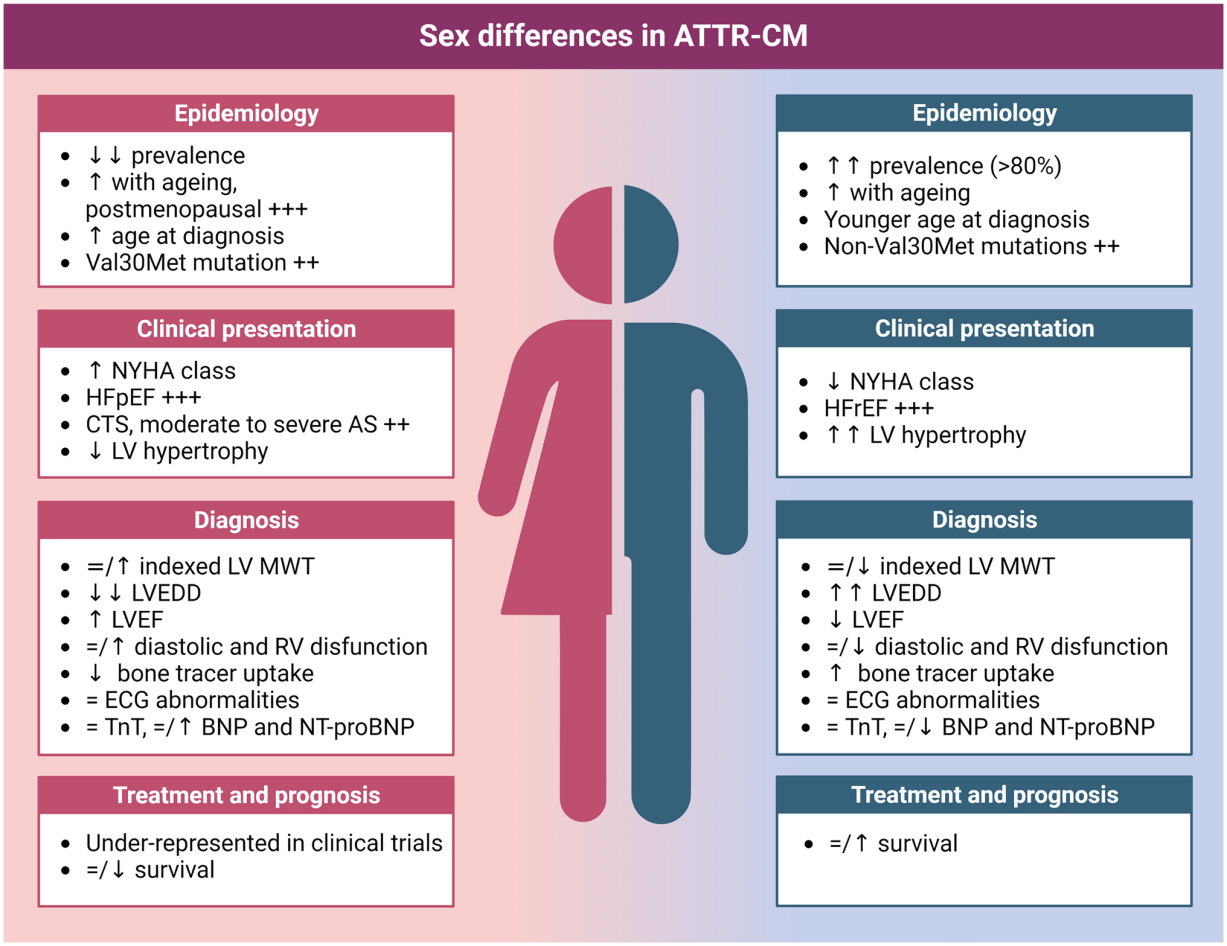
Sex differences in ATTR-CM

## Epidemiology

ATTR-CA may be either hereditary (variant ATTR-CA and ATTRv-CA) or due to amyloid deposition derived from wild-type TTR (wild-type ATTR-CA and ATTRwt-CA) [1]. In ATTRv-CA, an amyloidogenic mutation in the *TTR*gene, usually transmitted in an autosomal dominant fashion, facilitates the dissociation of its tetramer into monomers and promotes subsequent misfolding, whereas in ATTRwt-CA, the non-mutated, wild type TTR can cause amyloid formation in the presence of favorable conditions, such as aging and oxidative stress [4]. Autopsy studies have demonstrated that up to 20% of octogenarians died from heart failure had wild-type TTR amyloid deposits in the myocardium [5]. Despite this, ATTR-CA is still widely perceived as a rare disease and current estimates of its incidence and prevalence are scarce [6, 7]. However, over the last years, the use of bone scintigraphy for non-invasive diagnosis and novel disease-modifying therapies have prompted an active search for ATTR-CA [8].

Both ATTRwt- and ATTRv-CA predominantly affect men, with a prevalence increasing with age and accounting for > 80% of diagnosed cases [1]. Data from the Transthyretin Amyloidosis Outcomes Survey (THAOS), a global longitudinal survey on ATTR-CA, reported a striking male prevalence in 1.386 patients with ATTRwt-CA of 94% [3]. A meta-analysis conducted to estimate the sex distribution in patients with ATTRwt-CA has shown a male proportion of 86.9%, with a male to female ratio of 7:1. However, such proportion was significantly impacted by the age at diagnosis, with a male proportion of 69.5% or 92.7% in patients ≥ 80 years or < 80 years of age, respectively [9]. In a recent meta-analysis, the incidence and prevalence rate of ATTR-CA from 2000 to 2012 was respectively 17 per 100.000 person-years and 55 per 100.000 person-years, with a higher prevalence among men (70 per 100.000 person-years) compared with women (44 per 100.000 person-years) [10]. This difference may be attributed to different factors, including genetic and environmental factors, as well as diagnosis bias such as the lack of sex-specific cut-offs or the use of non-indexed parameters [11–13]. Moreover, in a recent meta-analysis on the epidemiology of ATTR-CA in different clinical settings, women accounted for 27% of patients with heart failure with preserved ejection fraction (HFpEF) and 36% of patients with carpal tunnel syndrome, two settings with a characteristic female preponderance, but also for 33% of patients with severe aortic stenosis [8]. These observations prompt further investigations on the biological relationship between sex and ATTR-CA.

Male predominance in ATTRv-CA is also well recognized. The Leu111Met, Ile68Leu, Thr60Ala, and Val122Ile mutations affect males in approximately 70% of cases [14–16]. A recent analysis from the THAOS showed that in 683 patients with ATTRv-CA, 493 (72.2%) were males, and 190 (27.8%) females [17]. However, there is growing evidence that the relationship between sex and ATTRv-CA calls into question age at diagnosis. Two recent national studies from the United Kingdom [18] and Spain [19] reported female sex to be associated with the presence of a pathogenic TTR mutation among patients aged ≥ 70 years old diagnosed with ATTR-CA. Reasons for the higher female prevalence of TTR mutations among older patients include the possibility of slower disease evolution, misdiagnosis, and/or the cardioprotective effect of estrogens during life [18]. Moreover, the THAOS registry likely does not reflect the full spectrum of ATTR-CA. Some geographical areas (and their endemic TTR variants) were indeed more represented than others, such as Portugal and the V30M variant. In addition, patients were diagnosed by cardiac biopsy, which is now restricted to selected case as ATTR-CA can be diagnosed noninvasively.

Women with ATTRwt-CA also have a higher age of onset than men [12, 20]. In the THAOS registry, both median age at enrolment (80 vs. 78 years; *p* = 0.002) and symptom onset (75 vs. 73 years; *p*= 0.045) were higher in female than male patients [3]. No significant differences were observed between sexes in symptom duration or time from symptom onset to diagnosis [3]. Similarly, in a Japanese study, women were significantly older at diagnosis (82.9 vs. 77.1 years, *p* < 0.001) and had a more advanced New York Heart Association (NYHA) functional class (2.6 vs. 2.2, *p*= 0.006) compared with men [21].

In conclusion, women are less affected by ATTR-CA and typically have older age at diagnosis. Further research is warranted to explore the underlying factors contributing to the sex differences in the epidemiology of ATTR-CA and to guide targeted diagnosis algorithm in both male and female populations.

## Pathophysiology

The mechanisms underlying TTR misfolding in ATTRwt amyloidosis are not fully understood, although deficits in proteostasis, proteolysis-induced fragmentation of TTR, environmental factors, and aging seem to play a key role [1]. Genetic factors play a critical role in the development and progression of ATTR-CA since pathogenic mutations in the *TTR*gene are known to cause hereditary forms of the disease. Interestingly, certain mutations exhibit sex-specific penetrance and phenotypic variations. Data analysis from the THAOS survey has shown that male prevalence is greater in the non-Val30Met cardiac mutations (Val122Ile, Leu111Met, Thr60Ala, or Ile68Leu) and in the Phe64Leu and Ile107Val mutations [17]. Conversely, the Val30Met mutation is more frequently found in females.

Sex hormones, including estrogen and testosterone, have been implicated in modulating cardiovascular physiology and pathology. Estrogen has been shown to exert protective effects on the cardiovascular system, including antioxidant, anti-inflammatory, and vasodilatory properties [22]. Moreover, 17β-estradiol has been shown to inhibit angiotensin II-induced cardiac hypertrophy and interstitial fibrosis [23]. In ATTR-CA, estrogen may confer a protective effect by reducing the detrimental effects of amyloid fibrils on cardiac function. Conversely, the decline in estrogen levels during menopause in females may contribute to disease progression and increased susceptibility to cardiac dysfunction. A study by Rapezzi et al. showed that women with ATTR-CA in the highest tertile of mean left ventricular (LV) wall thickness index were more often postmenopausal than those in the other two tertiles and had a much higher mean age; analogous age-related associations were not observable for men [24]. Sex hormones, both estrogens and androgens, may also directly influence TTR levels [25, 26]. 17β-estradiol, in particular, has been shown to induce TTR expression in murine choroid plexus via an estrogen receptor-dependent pathway [27]. Nonetheless, the role of estrogen signaling is still poorly understood and new evidence is emerging regarding the “estrogen paradox,” namely, in pulmonary arterial hypertension (PAH) [28]. In PAH, despite the pronounced tendency for the disease to develop in women, once affected by the disease, female patients exhibit better survival than men [29]. Further studies are needed to explore the molecular mechanisms of sex differences in cardiovascular diseases.

Amyloid plaques are made up of several different protein, for which sex differences in their composition exist. A proteomic analysis of amyloid plaques showed that males had higher abundance of QSOX1 and Serpine-2 but lower apolipoprotein A1 compared to females [30]. Further studies should evaluate whether such differences in plaque composition translate in different pathological mechanisms and outcomes.

Hypothetically, other mechanisms in ATTR-CA may exhibit sex differences; however, to the best of our knowledge, no clinical or preclinical studies have explored sex differences in such mechanisms to date. TTR misfolding and aggregation are central to the pathogenesis of ATTR-CA [31]. It has been suggested that chaperone proteins and proteostasis pathways may function differently in males and females, likely affecting the stability and degradation of amyloid fibrils [32]. Additionally, sex differences in autophagy and lysosomal function may influence the clearance of amyloid deposits [33]. The immune system plays a crucial role in the pathogenesis of ATTR-CA, with inflammatory processes and immune cell activation contributing to myocardial damage and fibrosis [34, 35]. Sex-based differences in immune responses and inflammatory signaling pathways may influence disease progression; generally, females exhibit a more robust immune response with greater antibody responses than males, higher basal immunoglobulin levels, and higher B cell numbers [36, 37].

Despite the scarce availability of studies, it seems likely to assume that the sex differences observed in clinical presentation and outcomes in ATTR-CA are explained by genetic, hormonal, and environmental factors.

## Clinical presentation

The clinical presentation and epidemiology of ATTR-CA has changed over time thanks to the introduction of non-invasive diagnostic tools, which allow earlier diagnosis and treatment [38]. Of note, most of the previously published studies on sex-differences included patients with biopsy-proven ATTR-CA, diagnosed in more advanced stages of cardiac disease. Sex-related differences in both clinical manifestations and red flags of ATTR-CA have been described [39]. In a study by Takashio et al., women were older at diagnosis and were more typically in class NYHA III (57% vs. 35%) or IV (7% vs. 1%) as compared to men [21]. The modified polyneuropathy disability (mPND) score is a measure of walking disability and ranges from 0 to IV; an analysis conducted on 1.386 patients with ATTRwt-CA from the THAOS showed that women had a larger proportion of IIIa or higher mPND scores than men (23% vs. 7%) [3].

Carpal tunnel syndrome is an established red flag for ATTR-CA, with a higher prevalence in females among the general population [40]. In a cohort of 98 patients (median age 68 years, 51% male) undergoing carpal tunnel release surgery, Congo red staining of tenosynovial tissue detected amyloid deposits in 6 males (60%) and 4 females (40%) [41]. Aortic stenosis is another typical finding in ATTR-CA. In a Japanese retrospective study, moderate to severe aortic stenosis was more frequently observed in women than men (45% vs. 5%; *p*< 0.001) [42].

Women with ATTR-CA typically present with HFpEF at diagnosis. In a prospective screening for ATTR-CA in 120 patients ≥ 60 years old (41% males) admitted due to HFpEF, 16 patients (13.3%) showed a moderate-to-severe uptake on the bone scintigraphy. Among these, 8 (50%) were women [43]. In a retrospective review conducted on 68 patients with ATTRwt-CA, the frequency of HFpEF was higher among females than males (85.7% vs. 61.0%, respectively; *p*= 0.025) [44]. Conversely, women typically present with a less evident hypertrophic phenotype [3, 21]. In a cohort of 298 consecutive patients (74% men) with increased LV thickness, 15 (5%) had ATTRv-CA (11 [73%] men) [45]. Similarly, a prospective study evaluated the prevalence of ATTR-CA among adult patients with an initial diagnosis of hypertrophic cardiomyopathy. Out of 343 patients, ATTR-CA was the most common unrecognized mimic (9% prevalence) and 198 (58%) were men [46].

All the studies above agree on the fact that ATTR-CA occurs later in life in females, with more frequently preserved ejection fraction and with a less hypertrophic phenotype.

## Diagnosis

Accurate and timely diagnosis of ATTR-CA is crucial for appropriate management and prognosis. However, emerging evidence suggests that sex differences may influence the diagnostic accuracy and interpretation of these imaging techniques and that females with ATTRwt-CA might be underdiagnosed, since the disease is thought to primarily affect elderly men. In a retrospective study of patients with ATTRwt-CA, those diagnosed post-mortem were more likely to be older and female than those diagnosed antemortem (31% vs. 9%, *p*≤ 0.001) [47]. Additionally, a post-mortem autopsy studies of patients with an ante-mortem diagnosis of HFpEF without clinically apparent amyloid reported a similar post-mortem rate of LV wild-type amyloid deposits among men (19%) and women (15%) [5]. Similar post-mortem autopsy studies from patients dying from any cause reported a prevalence of amyloid deposits in women equal or even higher than men [48, 49].

Echocardiography is widely used in the initial assessment and monitoring of patients suspected of having ATTR-CA. A nationwide Italian study recently explored the impact of echocardiography for orienting the diagnostic work up of amyloid disease [50]. Among a cohort of 5,315 consecutive patients ≥ 55 years old undergoing transthoracic echocardiogram for reasons other than CA, 381 had an echocardiogram suggestive for CA, and 62 were eventually diagnosed with CA (51 with ATTR-CA). In this study, there was no striking gender difference, but only a trend toward higher male prevalence was found among patients diagnosed with ATTR-CA compared to the other patients enrolled (63% and 52% males, respectively). Given the unselected nature of this cohort of patients, it could more likely reflect the real-word prevalence of ATTR-CA among the two sexes [50].

A LV wall thickness ≥ 12 mm plus at least one red flag should raise the suspicion of ATTR-CA. Nonetheless, since normal values of LV wall thickness are lower in women, the adoption of the same cut-off values for men and women could have contributed to the underdiagnosis or delayed diagnosis in women. A study by Aimo et al. on 330 patients with ATTR-CA (16% women) demonstrated that interventricular septum (IVS) and posterior wall (PW) thickness values were lower in women, as similarly reported by several previous studies [12, 51]. However, most differences were abolished when indexing IVS and PW by body surface area (BSA), height, or height^2.7^, suggesting similar disease severity when accounting for the smaller body size of women. PW thickness indexed for height^2.7^ was even higher in women [52]. Moreover, IVS values indexed by height^2.7^ displayed tighter associations with N-terminal pro-B-type natriuretic peptide (NT-proBNP), relative wall thickness, E/e' ratio, and tricuspid annular plane systolic excursion (TAPSE) values than non-indexed IVS values [52]. The authors therefore concluded proposing to replace the one-fit-all diagnostic cut-off of 12 mm with a specific cut-off for women based on the mean height of men and women in Europe, thus equal to 11 mm [52]. Similarly, a large study by Patel et al. on 1.732 ATTR-CA patients highlighted how body size significantly influences measures of disease severity. When indexed, overall structural and functional phenotype was similar between sexes [20]. A few significant differences suggested a mildly worse phenotype in females, characterized by greater indexed LV wall thickness, a greater E/e’ ratio, and a more severe degree of mitral and tricuspid regurgitation. When indexed for BSA, female patients were found to have a significantly greater mean IVS (9.62 vs. 8.88 mm/m [2], *p* < 0.001) and PW thickness (16.54 vs. 15.92, *p*= 0.001) than men; when indexed to height, mean PWT remained significantly greater in females but mean IVS was similar between sexes [20].

Sex-based differences are also observed in cardiac function and dimensions [20]. For instance, data from the THAOS demonstrated that female patients had a significantly higher mean LV ejection fraction (53% vs. 48%; *p* = 0.001) and lower mean LV diastolic diameter (42 vs. 46 mm; *p*< 0.001) than male patients [3], as confirmed by other studies [20, 21, 52]. A study by Zampieri et al. showed that in 259 patients with ATTRwt-CA (12% females), women had echocardiographic signs of more advanced disease at diagnosis. In particular, women had thicker indexed IVS (10 ± 1 vs. 9 ± 1 mm/m^2^, *p* = 0.009), higher diastolic dysfunction (E/e’ 25 [19–28] vs. 16 [13–20], *p* = 0.03), and worse right ventricular function (TAPSE 15 ± 4 vs. 17 ± 4 mm, *p*= 0.04) than men [53].

CMR provides detailed information on myocardial tissue characteristics, including late gadolinium enhancement (LGE), extracellular volume (ECV), and T1 mapping, which are valuable for diagnosing, prognosing, and assessing the extent of cardiac amyloid deposition in ATTR-CA [54]. However, no data are currently available regarding sex differences in CMR-assessed parameters. Bone scintigraphy has revolutionized the non-invasive diagnostic algorithm for ATTR-CA [11]. It is interesting to note that it seems there are sex differences even in the bone tracer cardiac uptake. In the study by Takashio et al., the mean heart-to-contralateral ratio obtained using 99mTc-labeled pyrophosphate (99mTc-PYP) was significant lower in women (1.64 vs. 1.89; *p* = 0.001).

In addition to imaging modalities, biomarkers play a key role in the diagnosis and management of ATTR-CA. While troponin levels appear to be similar between sexes, contrast evidence exists on NT-proBNP levels. Some studies report similar NT-proBNP levels in women and men, whereas some others show higher NTproBNP [53] and BNP [21] values in women at diagnosis. Age of onset, comorbidities, and the type of population studied could explain such different findings.

Electrocardiographic red flags of ATTR-CA include atrial fibrillation, atrioventricular or bundle branch blocks, low voltages, and a pseudo-infarct pattern. Apparently, there are no electrocardiographic differences between sexes in electrocardiographic findings [3, 21, 53].

To summarize, women with ATTR-CA may have been misdiagnosed and under-diagnosed. Despite an apparent minor wall thickness, when indexing for BSA or height, women show equal or even higher LV thickness than men. Moreover, they show a smaller LV cavity and a more preserved ejection fraction. No clear differences are seen or available at ECG and CMR, as well as in biomarker levels.

## Treatment

For a long time, the only therapy available for ATTR amyloidosis was liver transplantation or combined liver–heart transplantation. As of 2023, tafamidis is the only medication approved by both the U.S. Food and Drug Administration (FDA) and European Medicines Agency for the treatment of ATTR-CA, both variant and wild type [11, 55]. It is a disease-modifying drug acting as a TTR stabilizer, thus inhibiting the dissociation of TTR with fibril formation and cardiac deposition [56]. Female sex, low disease severity, and high native TTR concentration at the initiation of treatment predicted a positive response to tafamidis in ATTRv-polineuropathy (PN) [57]. The same cannot be said for ATTR-CA; studies conducted on the safety and efficacy of tafamidis mainly included male patients with no further evaluations on different response between male and female patients [58]. In a pre-specified analysis of 335 patients (only 30 females) from ATTR-ACT (Tafamidis in Transthyretin Cardiomyopathy Clinical Trial), the aim was to determine the effect of tafamidis vs placebo between ATTRv- and ATTRwt-CA. All-cause mortality and change from baseline to month 30 in 6-min walk test distance and Kansas City Cardiomyopathy Questionnaire Overall Summary score were compared in patients with ATTRwt-CA and ATTRv-CA. The reduction in mortality and functional decline with tafamidis treatment was similar in both disease subtypes and with no sex-related differences [59]. Moreover, a Japanese single-center retrospective study examined 125 patients with ATTRwt-CA treated with tafamidis and 55 untreated patients. The results showed that female sex was not a significant predictor for composite clinical outcomes in patients undergoing tafamidis treatment [21].

Second-generation TTR stabilizer acoramidis (AG10) determined a rise of stable tetrameric TTR levels up to 51% in a phase II trial conducted on 49 symptomatic patients with transthyretin amyloid cardiomyopathy [60]. AG-10 is being evaluated in a phase III trial on patients with ATTR-related cardiomyopathy (ATTRibute-CM, NCT03860935).

Recently, new pharmacological therapies have entered clinical practice and some others have been evaluated in preclinical studies [61].

Reducing or eliminating TTR expression with so-called TTR silencers, antisense oligonucleotides (ASOs) and small interfering RNA (siRNA), is one approach that slow down the progression of ATTR-CA, both binding TTR mRNA and inducing subsequent degradation [62]. A first-in-class ASO for TTR amyloidosis, inotersen, was evaluated in the NEURO-TTR trial in 172 patients with familial amyloid polyneuropathy with or without ATTR-CA. Patients receiving inotersen had a mean reduction of 74% of TTR serum levels reaching a steady state by week 13. In the NEURO-TTR trial, no sex differences in response to inotersen were observed, as assessed by Norfolk QoL-DN or mNIS + 7 questionnaire score [63]. AKCEA-TTR-LRx, a new-generation ASO, was evaluated in the preclinical and phase I ION-682884-CS1 study with a greater than 85% reduction in TTR serum levels in ATTR-CA patients without the adverse side effects reported in the NEURO-TTR trial [64].

siRNAs represent the other way to reducing TTR expression, after their inclusion into an hepatocytes RNA-induced silencing complex (RISC), binding the target mRNA to facilitate its degradation and prevent its expression. In a phase-II clinical trial in 2015, patisiran reduced mean TTR serum levels by approximately 80%. Reductions in LV wall thickness (∼1 mm) and NT-proBNP levels (∼55%) were observed in patients treated with patisiran in the phase III study APOLLO [65]. Both patisiran and inotersen are approved for the treatment of patients with ATTR-familial amyloidotic PN.

CRISPR (clustered regularly interspaced short palindromic repeats)/Cas9 gene editing technology represents a new silencing prospective for ATTR-CA that is able to reduce TTR levels. Finn et al. demonstrated that a single dose of LNP-INT01 was able to reduce serum levels of TTR by greater than 97% for at least 12 months [66].

A phase I study was designed to evaluate the safety of PRX004, a humanized monoclonal antibody able to target and clear the non-native transthyretin aggregates (misTTR) associated with disease pathology that underlies both ATTRwt- and ATTRv-CA, without affecting the native, or normal tetrameric form of the protein. Among the seven patients with cardiac involvement, the authors reported an improved global longitudinal strain, even if the study has been beforehand terminated because of the COVID pandemic [67]. NI006, a recombinant human anti-ATTR antibody, has been recently evaluated in a phase I trial showing good safety, with no apparent drug-related serious adverse events. Furthermore, cardiac tracer uptake on scintigraphy and extracellular volume on cardiac magnetic resonance imaging, median NT-proBNP, and troponin T levels appeared to be reduced over a period of 12 months [68].

Despite the multiple therapeutic strategies, as already mentioned, none of the trials mentioned analyzed the different pharmacokinetics and pharmacodynamics according to gender. Moreover, women have been severely under-represented in the clinical trials conducted so far (Table 1). Safety and efficacy outcomes could differ in female patients due to their smaller body surface area, tendency to be older at the moment of diagnosis, and with a better cardiac phenotype. New studies are expected in this sense, for the field of a gender-related cardiology not only on the diagnostic aspect but also on the therapeutic one.

**Table 1:** Women representation in ATTR-CM phase 2 and 3 trials

	**Trial**	**Drug**	***n*** **m (%)/f (%)**	**Sex differences in treatment response**	**Reference**
**Tetramer stabilizers**	ATTR-ACT Phase 3 (NCT01994889)	Tafamidis	441 398 (90.3)/43 (9.7)	N/A	Maurer et al. [56]
	Study of AG10 in Amyloid Cardiomyopathy Phase 2 (NCT03458130)	Acoramidis	49 45 (92)/4 (8)	N/A	Judge et al. [60]
**RNA silencers**	APOLLO Phase 3 (NCT01960348)	Patisiran	225 167 (74.2)/58 (25.8)	N/A	Adams et al. [65]
	NEURO-TTR Phase 3 (NCT01737398)	Inotersen	172 118 (68.6)/54 (31.4)	No significant differences at Norfolk QoL-DN and mNIS + 7 scores	Benson et al. [63]
	CARDIO-TTRansform (*ongoing*) Phase 3 (NCT04136171)	Eplontersen	1400^a^ N/A / N/A	N/A	N/A
	HELIOS-B (*ongoing*) Phase 3 (NCT04153149)	Vutrisiran	655^a^ N/A / N/A	N/A	N/A
	ENDEAVOUR Phase 3 (NCT02319005)	Revusiran	206 158 (76.7)/48 (23.3)	N/A	Judge et al. [60]
**Deposits clearance agents**	A Study of Doxycycline and TUDCA Plus Standard Supportive Therapy Versus Standard Supportive Therapy Alone in Cardiac Amyloidosis Caused by Transthyretin (*ongoing*) Phase 3 (NCT03481972)	Doxycycline and tauroursodeoxycholic acid	102 N/A / N/A	N/A	N/A

^a^Last updated record as of 06/18/2023. *ATTR-ACT *Tafamidis in Transthyretin Cardiomyopathy Clinical Trial, *f *females, *m* males, *mNIS* modified neuropathy impairment score, *N/A* not available, *QoL-DN* quality of life for diabetic neuropathy, *TUDCA* tauroursodeoxycholic acid

## Outcome

Multiple factors may influence the prognosis of ATTR-CA. The specific genetic mutation plays a significant role, since certain mutations have been associated with a more aggressive disease course [69]. The extent of cardiac involvement, including the severity of myocardial infiltration and functional impairment, also affects prognosis [51]. Additional comorbidities, such as renal insufficiency or diabetes, can further impact patient outcomes [21, 69]. However, few studies so far examined prognostic differences based on sex.

Overall, recent studies have not demonstrated any significant differences between sexes in terms of overall prognosis, even if a certain level of myocardial protection seems to be present in female patients in premenopausal status [24].

## Conclusions and future perspectives

Understanding the impact of sex differences in ATTR-CA is of paramount importance for accurate diagnosis and management of this condition. However, there are notable challenges that need to be addressed. The true prevalence of ATTRwt-CA may have underestimated in women due to a sex-related bias in identifying the condition, which has also been observed in other cardiovascular diseases [69]. The reliance on non-indexed wall thickness measurements may have resulted in the underestimation of affected females and subsequent delays in diagnosing the condition [51]. Sex-specific cut-off values are essential to account for physiological sex differences in cardiac structure and function, thus allowing an accurate and timely diagnosing and management of ATTR-CA. The under-representation of women in clinical trials may have also limited the evidence on sex-specific management and outcomes.

Efforts should be therefore made to promote inclusion of women in clinical trials, ensuring adequate representation and allowing for sex-specific analysis. Moreover, updating guidelines to include indexed thickness values and incorporating sex-specific cut-off values in diagnostic algorithms would improve the accuracy of diagnosing ATTR-CA in women. By addressing these challenges, we can advance our understanding of sex differences in ATTR-CA and pave the way for more personalized and effective management strategies for both men and women affected by this condition.

## Data Availability

Not applicable.
